# Positive and Existential Psychology in Times of Change: Towards Complex, Holistic, Systemic, and Integrative Perspectives

**DOI:** 10.3390/ijerph19148433

**Published:** 2022-07-10

**Authors:** Claude-Hélène Mayer

**Affiliations:** Department of Industrial Psychology and People Management, College for Business and Economics, University of Johannesburg, Johannesburg 2006, South Africa; claudemayer@gmx.net

## 1. Introduction

This Special Issue, entitled “Positive and Existential Psychology in Times of Change: Towards Complex, Holistic, Systemic and Integrative Perspectives”, addresses the urging questions and challenges of our times from the latest positive and existential psychology perspectives. Thereby, the authors aim to publish the latest trends from theoretical, empirical, and applied perspectives, and contribute from various disciplinary, cultural, and language backgrounds.

During the past two decades, positive psychology (PP1.0) has been pioneered by Seligman [1,2] and Seligman and Csikszentmihalyi [3], who shifted the focus of research from a rather negative towards a more positive perspective. Since then, the positive psychology movement has gained in importance [4,5,6].

Wong, P.T.P. [7,8] developed this movement further to a so-called positive psychology wave 2 (PP2.0) or Existential Positive Psychology (EPP) perspective. He highlighted that one can only develop their positive side after having worked through the negative aspects of life, such as suffering and pain [9]. Wong’s concept is dualistic as well as systemic in nature, taking different system’s elements into consideration. PP2.0 or EPP have become important terms and concepts in the positive psychology movement [10,11,12,13,14]. Moreover, researchers such as Lomas et al. [15] have since called for the introduction of a third wave of positive psychology (PP3.0), which would ask questions with a view to a more systemic understanding of the development of positive psychology concepts.

In this Special Issue, it is argued that an existential positive psychology (EPP) is needed in theory as well as in applied practice—such as therapy, counseling, and consulting—which deals with the complexity of life, responding to essential questions of our times, providing adequate answers. This Special Issue addresses the key components of EPP from a holistic, systemic, and integrative point of view. Key questions of this Special Issue include interdisciplinary and intercultural insights into management, education, crime studies, mental health, and children’s studies in different cultural contexts, which will be introduced briefly in the following section.

## 2. Articles in This Special Issue

The first article by Claude-Hélène Mayer, Cemonn Wegerle, and Rudolf M. Oosthuizen refers to the stress individuals have experienced during COVID-19 and how managers have coped with the necessitated changes during COVID-19 from a positive psychology and salutogenic perspective. They present a qualitative study which shows that managers have a complex world view. The findings demonstrate the complex connections of high/low SOC scores and the managers’ explorations and systemic understanding regarding their managerial world.

In this Special Issue, Sharon Johnson and Izanette van Schalkwyk write about “Bridging the gap of the Afri-Eurocentric Worldview divide in a postcolonial South Africa”. The authors attempt to bridge the gap between Africentric and Eurocentric worldviews through the lens of positive psychology’s second wave of attaining pathways to well-being through a qualitative methodological approach. The researchers found that students’ worldviews were shaped by their primary caregivers’ multicultural influences, as well as their exposure to educational and religious contact zones. Positive psychology offers a space to accommodate well-being as a healing process, not only for the oppressed, but also the oppressors of historical social injustices.

Claude-Hélène Mayer explored “Understanding wildlife crime from Eco-Existential and African Perspectives: A Psycho-Philosophical Investigation”. The author explains how human–wildlife interactions are influenced by underlying existential “givens” and culture-specific aspects that need to be investigated to understand why wildlife crime is on the rise. This theoretical article explores (eco-)existential perspectives, Greening’s four givens, and selected African philosophical concepts, aiming to understand the complexities behind the prevalence of wildlife crime within global and African contexts.

Kathryn Anne Nel and Saraswathie Govender explore the question as to what has encouraged individuals to carry on with life during the global COVID-19 crisis. The authors discuss the writings of Wong in their conceptual paper, who worked within the framework of EPP, Frankl, a holocaust survivor, whose work falls within the scope of humanistic and existential psychology and Asante’s Afrocentrism, which is a philosophical framework grounded on the African continent.

Michelle May writes about the Robben Island diversity experiences (RIDEs), a conference using Tavistockian Technology, also known as Group Relations. The author explores the conferences held at Robben Island in a long-term study from 2000 to 2013 in terms of the unconscious and covert South African diversity dynamics which are studied as they manifest among managers and officials in the fields of change, diversity management, transformation, and human resources management. The study provides interesting insights into changing racial hierarchies, group relations, and leadership, as well as the marginalization of women. The findings can be used by researchers, consultants, and managers to enhance the understanding of diversity dynamics in contemporary South African organizations.

The subsequent article waas written by Catherine Malboeuf-Hurtubise, Carina di Tomaso, David Lefrancois, Geneviève A. Mageau, Geneviève Taylor, Marc-André Their, Mathieu Gagnon and Terra Léger-Goodes, and is entitled “Existential Therapie for Chiildren: impact of a Philosophy for Children Intervention on Positive and Negative Indicators of Mental Health in Elementary School Children”. The goal of the study was to evaluate the impact of P4C on basic psychological need satisfaction and mental health in elementary school students. The authors present quantitative findings, showing that P4C may be a promising intervention to foster greater autonomy in elementary school children, while also improving mental health.

Finally, an international group of authors published a paper on “Self-Compassion in Irish Social Work Students: Relationships between Resilience, Engagement and Motivation”. This study aimed to evaluate (i) relationships between self-compassion and more traditional positive constructs—resilience, engagement, and motivation—and (ii) differences in these constructs between the levels of studies to inform how self-compassion can be enhanced in social work students. The authors suggest that social work educators across different levels can strengthen students’ resilience and intrinsic motivation to cultivate students’ self-compassion.
